# Emotional and behavioural difficulties among children and adolescents attending “ART teen clubs” in Mzuzu City in northern Malawi: a cross-sectional study

**DOI:** 10.1186/s12887-023-04504-1

**Published:** 2024-01-13

**Authors:** Paul Uchizi Kaseka, Maggie Zgambo, Balwani Chingatichifwe Mbakaya, Mathews Lazarus, Obed Nkhata, Fatch W. Kalembo

**Affiliations:** 1 Paediatric Department, Mzuzu Central Hospital, Private Bag 209, Mzuzu, Malawi; 2 School of Nursing and Midwifery, Edith Cowan University, Joondalup, Western Australia; 3 School of Nursing, Curtin University, Bentley, Western Australia; 4 Public Health, University of Livingstonia, Mzuzu, Malawi; 5 Nursing and Midwifery Department, Mzuzu University, Mzuzu, Malawi; 6 Basic Department, Kamuzu University for Health Sciences, Lilongwe, Malawi; 7 Investigations Department, Nurses and Midwives Council of Malawi, Mzuzu, Malawi

**Keywords:** Children and adolescents living with HIV, Emotional and behavioural difficulties, ART Teen Club, Malawi

## Abstract

**Background:**

While triple anti-retroviral therapy (ART) has improved HIV-infected children surviving into adolescence and adulthood, these children remain vulnerable to HIV-related psychological disturbance due to both the direct HIV infection effects on the brain and indirect effects related to coping with a range of medical, psychological and social stresses associated with HIV, which makes it vital to identify their mental health needs. This study assessed the emotional and behavioural challenges of HIV perinatally infected children and adolescents with a completed disclosure process attending “ART teen club” in Malawi.

**Methods:**

A cross-sectional descriptive study design was conducted to obtain quantitative descriptive descriptions of emotional and behavioural challenges among HIV-infected children and adolescents between 10 and 22 years of age. They were interviewed on their family socio-demographic characteristics, clinical characteristics as well as emotional, conduct, hyperactivity, peer and prosocial problems using the Chichewa version of the Strengths and Difficulties Questionnaire. Data were analyzed using descriptive analysis and logistic regression.

**Results:**

Based on the four-band categorization of the SDQ, higher scores for total difficulties score were observed in 72.9% of the children. According to the subscales of the SDQ, results show that children had higher scores in peer problems (62.8%), emotional (68.2%), conduct (68.6%) and prosocial (57.8%) subscales while lower scores were identified in the hyperactivity (16.6%) subscale. Results show that within each level, males are having lower frequencies as compared to females. Results from multivariate binary logistic regression indicate that those with a single parent or not as well as the WHO HIV clinical stage had an impact on the mental health status of the children. Children who do not have a single parent (AOR 3.404; 95% CI:1.563–7.416, *p* = 0.002) had 3.404 odds of having abnormal mental health status unlike those children with a single parent and children who were in WHO HIV clinical stage 2 (AOR 2.536; 95% CI:1.005–6.395, *p* = 0.049) or 3 and 4 (AOR 8.459; 95% CI:1.5.820-10.544, *p* < 0.001) had more odds of having the mental disorder as compared with those children in WHO HIV clinical stage 1.

**Conclusion:**

The findings of this research underscore the multifaceted nature of mental well-being among children and adolescents living with HIV. Elevated scores in total difficulties, emotional, conduct, and peer problems signify areas of concern, while disparities in hyperactivity and prosocial behavior highlight the nuanced nature of their behavioral challenges. Recognizing the inadequacy of a one-size-fits-all approach, the research emphasizes the necessity of a comprehensive strategy, incorporating factors like religious background, family structure, and clinical HIV stage. Furthermore, the role of “ART teen clubs” in this context is pivotal. Beyond addressing identified risk factors, these clubs must actively foster resilience. Creating an inclusive environment, tapping into individual strengths, and nurturing a sense of community are vital components. By adopting such a holistic approach, Teen support clubs can significantly contribute to the overall mental well-being of adolescents living with HIV, enabling them to navigate challenges effectively and thrive amidst their circumstances.

## Introduction

In 2021, an estimated 38.4 million people were living with Human Immunodeficiency Virus (HIV) globally, and of these, 2.73 million were children and adolescents below 19 years [1]. The majority of children and adolescents living with HIV are in sub-Saharan Africa. Similarly, in 2020, sub-Saharan African countries accounted for about 89% of new HIV paediatric infections. Malawi is one of the countries worst burdened by HIV pandemic and HIV/AIDS remains a major public health problem in Malawi despite a significant reduction in the number of new infections in recent years [1]. In 2020, approximately, one out of 18 million people were living with HIV in Malawi, and approximately 6% of those affected were children under the age of 15 years [1].

HIV/AIDS is the second leading cause of death among adolescents aged 10–19 years globally and in sub-Saharan Africa [2] partly due to inadequate HIV testing and counselling and substandard follow up for HIV infected children and adolescents on antiretroviral therapy (ART), causing higher mortality and morbidity rates [3]. For example, out of 650,000 people who died of AIDS-related illnesses globally in 2021, 110,000 (17%) of them were children and adolescents under 20 years of age [4]. This situation needs curbing to prevent worsening, especially with the prolonged COVID-19 pandemic, global economic crisis, overloaded health care systems and constrained access to life-saving services [5].

While the provision of triple ART has increased the life expectancy of children and adolescents living with HIV, these children are at risk of poor developmental outcomes due to both the direct and indirect impacts of HIV infection [6]. One of the impacts of HIV infection is psychosocial problems, which intensify in adolescence or early adulthood. In addition to trying to navigate the world while living with HIV, these children and adolescents grow to deal with social pressures associated with adolescence for emotional regulation and social development [7, 8]. Therefore, children and adolescents living with HIV need support to have a positive sense of identity, manage thoughts and emotions, build social relationships, acquire education, and to actively integrate in the society [9].

Authors of systematic reviews have reported high rates of common mental disorders among children, adolescents and adults living with HIV in sub-Saharan Africa [10–12]. The most common mental health disorders among children and adolescents in sub-Saharan Africa are attention deficit hyperactivity disorder (ADHD), mood, depression, anxiety, conduct problems, violent behaviour, and emotional and behavioural problems [6, 13, 14]. Previous studies have shown that mental and behavioural health challenges are prevalent in HIV-infected adolescents and pose an enormous burden than HIV-negative peers especially in resource-limited settings [11, 15]. Besides, these children may experience neurocognitive complications like deficits in cognitive, speech, gross motor and fine motor functioning which can greatly impact their social relationships, academic achievements, general health, and risk of substance abuse [16–18]. Authors of recent studies have found that these cognitive deficits persists, despite early initiation of ART and viral suppression [19, 20].

Among HIV positive children and adolescents, mental health problems have serious implication for later physical health through its impact on health-related behaviour such as smocking, substance abuse, unsafe sex and non-adherence to medication which are detrimental to their health [21]. To prevent these issues there is need to have a differentiated ART service delivery model with tailored, layered, and combined prevention packages that are age-appropriate offered in venues that are acceptable and patronized by the adolescents [22].

To meet the unique needs of the children and adolescents living with HIV and thereby increase treatment adherence and achieve viral suppression, the Malawi Ministry of Health, uses a Teen Club model which was adapted from the Baylor College of Medicine International Paediatric AIDS Initiative-Centre of Excellence Curriculum [23]. The “ART teen club” program, a peer support group for adolescents living with HIV, is a globally recognized model that empowers adolescents to build positive relationships, improve self-esteem and ultimately improve both clinical and mental health outcomes [24]. The “ART teen club” is a targeted psychosocial support intervention which uses different strategies to address the barriers faced by adolescents living with HIV to achieve optimal treatment outcomes [25]. The “ART teen club” mission is to provide a safe and nurturing environment for HIV-infected adolescents to build supportive relationships, increase their self-esteem and develop and reinforce good habits [24]. According to MacKenzie, Lettow, Gondwe et al., 2017 at “ART teen clubs”, teens do ART refills, are assessed for adherence, and are provided with individualized peer counselling and support as necessary. These children and adolescents are also stratified by age and gender into small group sessions for sexual and reproductive health education sessions in order to encourage comfort and privacy. In addition, these teens participate in recreational activities like facilitated sports, arts and games for peer interaction [25]. “ART teen club” provides a forum to address other issues affecting HIV-infected adolescents, such as nutrition and other psychological needs. Adolescents are eligible for participation in the “ART teen club” if they are between the ages of 10 and 19, on ART, and have had their HIV status disclosed to them. These adolescents are referred to the Teen club by either the ART clinician or a nurse after the HIV disclosure process has been completed. Teen club sessions are held monthly on a weekend (usually Saturday) to avoid school absenteeism which is one of the challenges to HIV treatment adherence amongst adolescents in low socio-economic settings like Malawi [23, 26]. Teens are supposed to graduate to adult ART clinics after their 19th birthday. The adolescents recruited in this study were in “ART teen clubs” run by regular ART clinicians and nurses who have been trained in the paediatric HIV care curriculum. These health workers work on a roaster and are compensated accordingly for the extra hours in line with the Ministry of Health or non-governmental organization (NGO) policies.

Studies on the teen club model have shown that the model is more effective towards attaining virological suppression among adolescents living with HIV in Malawi [25, 27]. However, there is a dearth of knowledge on the impact of this model on mental wellness of its consumers. This population is particularly vulnerable to mental health and conduct problems because of age, HIV diagnosis and poor social economic factors, which if left unidentified or treated could persist to adulthood [28]. Understanding the emotional and behaviours difficulties among this population has a potential to provide valuable information on the subject to policy makers and first line health workers. This information can be used to develop polices and interventions that can better support the need of adolescents living with HIV. We, therefore, conducted the study to establish the prevalence of emotional and behavioural problems among children and adolescents living with HIV. Specifically, the study had two research questions namely (1) What is the prevalence of emotional and behavioural difficulties among children and adolescents attending ART teen club and (2) What demographic and clinical factors are associated with emotional and behavioural problems among this population? Identifying the prevalence of emotional and behavioural problems among the study sample provides a baseline understanding of the scope and magnitude of the issue within the target population. If a significant portion of the population is affected, it signals the need for interventions on a larger scale. Understanding the demographic and clinical factors associated with emotional and behavioural difficulties helps in identifying high-risk groups within the sample. For instance, if a certain demographic group or those with specific demographic characteristics are more prone to these problems, interventions can be targeted towards these groups. In addition, resources for interventions are often limited. Understanding the associated factors helps in efficient resource allocation, directing efforts towards the factors that have the most significant impact on emotional and behavioural difficulties in this population.

## Methods

### Research design, study population and recruitment criteria

This was cross-sectional study conducted with children and adolescents. We targeted children and adolescents living with HIV in Mzuzu city. Potential participants were recruited in the study if they were: (1) living in Mzuzu and enrolled in the “ART teen club”; (2) aware of their HIV status; (3) had informed consent to participate in the study. Since all of them were below 18 years old, their parents signed informed consent forms for them to participate in the study. We planned to exclude children and adolescents who had an illness with a potential to impact on informed consenting process and completion of the study questionnaire such as psychiatric and severe HIV related illnesses. However, none was excluded based on these criteria.

### Research setting

This study was undertaken in three “ART teen clubs” each at Mzuzu Central Hospital, Mzuzu Health Centre and St John’s Mission Hospital in Mzuzu City, Northern Malawi. All these facilities provided HIV services to teens one weekend in the month. Mzuzu Central Hospital ART teen clinic has a cumulative total of 324 clients, Mzuzu Health Centre 195 clients and St John’s Hospital 184 Clients. These sites were purposively selected for this study because they were the only facilities with “ART teen clubs” in Mzuzu City.

### Sampling and sample size

Participants to this study were recruited through systematic sampling between April and May 2022. We used an online sample size calculator (Raosoft, 2020) to determine the required sample size for this study [29]. We found that the three health facilities had in total 703 children and adolescents enrolled in “ART Teen Clubs.” Based on the estimated population, and setting the margin error and confidence level at 0.05 and 95% respectively, a sample size of 249 children and adolescents was needed. To include a non-response rate of 15% a total sample size of 286 was considered adequate.

### Data collection instruments

Data were collected using a questionnaire that had two sections: (1) Socio demographic variables and (2) child emotional and behavioural difficulties. The sociodemographic sections contained questions about age, gender, educational level of parents/primary caregivers, and availability of parents. The child emotional and behavioural difficulties was assessed by the youth version of the Strengths and Difficulties Questionnaire (SDQ-Y) [30]. The SDQ-Y is a self-report tool, designed to be completed by children and adolescents aged 11–17 years and used to detect childhood emotional and behavioural problems. The SQD-Y is a well-validated instrument. The scale has adequate internal consistency (Cronbach’s alpha ranging from 0.78 to 0.82) and predictive validity [30]. This questionnaire has been used in more than 50 studies across 12 African countries including Malawi [31] and is translated in 60 different languages including Chichewa, the Malawian local national language [32]. The tool has 25 items divided in five subscales comprising of five domains of conduct problems, hyperactivity/inattention, emotional symptoms, peer problems and prosocial behavior. While four of the subscales refers to problem behaviours, the fifth (prosocial behaviour) refers to positive behaviour. We also collected data from participants clinical records on HIV disclosure status, and WHO clinical stage of HIV.

Data were collected by research assistants with a Bachelor of Science in Nursing qualification. The research assistants were trained in the data collection process. We used the original Chichewa-translated version of the SDQ-Y to collect data from all participants. For participants who were illiterate, the research assistants read the questions to the participants and marked the answers selected by the participants on the questionnaire.

### Recruitment and data collection procedures

Potential participants were assigned numbers from one to last person based on the time they arrived at the “ART teen club”. Those with even numbers were screened based on the inclusion criteria (see Sect. 2.1 above). Research assistants with a degree or diploma in Nursing were recruited to assist with data collection. These research assistants were trained on the research procedure prior to data collection. In March, 2022, our research assistants, who were not part of the teen club staff, informed potential participants about the aims, procedure, outcomes, benefits, and associated risks as well as their rights with regard to consent, confidentiality, anonymity and withdrawal from the study. The research assistant further distributed the informed consent/assent forms and study information sheets to potential participants. Participants brought their signed informed consent forms to the clinic on their next visit. The research assistants confirmed the understanding of the study purpose, reminded participant that of voluntary participation, assured participant of their confidentiality and verified if non coerced consenting was made prior to data collection. Participants were asked to complete their questionnaires in a private room at the clinic soon after clinic activities. Data collection took place between April and May 2022. Completion of the questionnaire took approximately 20 min.

### Ethical consideration

This research was granted ethical approval by the Malawi National Health Sciences Research Committee (Ref: #17/05/1804). Written permission to conduct the study was obtained from three participating facilities. The participants were informed that they had the right to withdraw from the study at any time without any prejudice. All participants provided consent/assent prior to data collection.

During the research process, respondents were not subjected to any physical harm as the study did not involve any invasive procedures. Prior arrangements for counselling and support were made for anticipated emotional reactions by the participants considering the emotional nature of the topic under study. The respondents answered the questions in a comfortable environment with privacy assured and no public interference.

### Statistical analysis

Descriptive statistics were used to understand the general characteristics of participants. Bivariate analysis was conducted using binary logistic regression to identify individual factors that are associated with emotional and behavioural difficulties. All variables that were significant at *P* ≤ 0.05 in bivariate analysis were entered into multivariate logistic regression to identify factors that were independently associated with emotional and behavioural difficulties among the study population. All analyses were considered statistically significant at *P* ≤ 0.05. Data analyses were conducted using statistical Package for Social Sciences (SPSS) version 22.

## Results

### Response rates and demographic characteristics

A total number of 286 questionnaires were distributed and 277 were returned resulting in 96.8% response rate. This study recruited participants whose ages ranged from 10 to 17 years with a mean of 14 years. The majority of the participants were female (71.5%, n = 198) and recruited from three health facilities in Malawi of which majority came from Mzuzu Central Hospital (46.9%, n = 130) and the least came from St John’s Hospital (18.8%, n = 52). Results further indicate that the majority of children sampled were doing their studies at primary school followed by those who were at secondary school. Regarding parents’ education, 70.3% of children in the study had parents who had secondary education or above with only 10% whose parents had no formal education. Among the sampled children, the majority were protestants (49.5%, n = 137) and 51.6% of them had both parents. The household size of the sampled children ranged from 2 to 11 individuals with a mean of 5 individuals per household (See Table 1).

**Table 1:** Demographic characteristics of the participants

Demographic Characteristics	Frequency	Percent
**Sex**		
Male	79	28.5
Female	198	71.5
**Health Facility**		
Mzuzu Central Hospital	130	46.9
Mzuzu Health Centre	95	34.3
St John’s Hospital	52	18.8
**Participant’s level of education**		
None	5	1.8
Primary	153	55.2
Secondary	110	39.7
Tertiary	9	3.2
**Guardian/parent’s level of education**		
None	10	3.6
Primary	72	26
Secondary	145	52.3
Tertiary	50	18
**Guardian/parents occupation**		
Unemployed	31	11.2
Informal employed	30	10.8
Formal employed	79	28.5
Business	115	41.5
Others	22	7.9
**Participant’s religion**		
Protestant	137	49.5
Roman Catholic	53	19.1
Pentecostal	48	17.3
Traditional	14	5.1
Muslim	19	6.9
Apostolic	6	2.1
**Availability of both parents**		
Yes	143	51.6
No	134	48.4

### Prevalence of emotional and behavioural difficulties of participants

Basing on the four-band categorization of the SDQ, higher scores for the total difficulties scale score was observed in 72.9% of the children. According to the subscales of the SDQ, results show that children had higher scores in emotional (68.2%), conduct (68.6%), peer problems (62.8%) and prosocial behaviour (57.8%) subscales while a lower score was identified in the hyperactivity (16.6%) subscale (see Table 2).

**Table 2:** Psychosocial characteristics of the study participants

Psychosocial characteristics	Frequency	Percent
**Total Difficult Scoring**		
Close to average	75	27.1
Slightly raised/high/very	202	72.9
**Emotional Problem Scoring**		
Close to average	88	31.8
Slightly raised/high/very	189	68.2
**Conduct Problem Scoring**		
Close to average	87	31.4
Slightly raised/high/very	190	68.6
**Hyperactivity Scoring**		
Close to average	231	83.4
Slightly raised/high/very	46	16.6
**Peer Problem Scoring**		
Close to average	103	37.2
Slightly raised/high/very	174	62.8
**Prosocial Scoring**		
Close to average	117	42.2
Slightly lowered/low/very	160	57.8

**Fig. 1:**
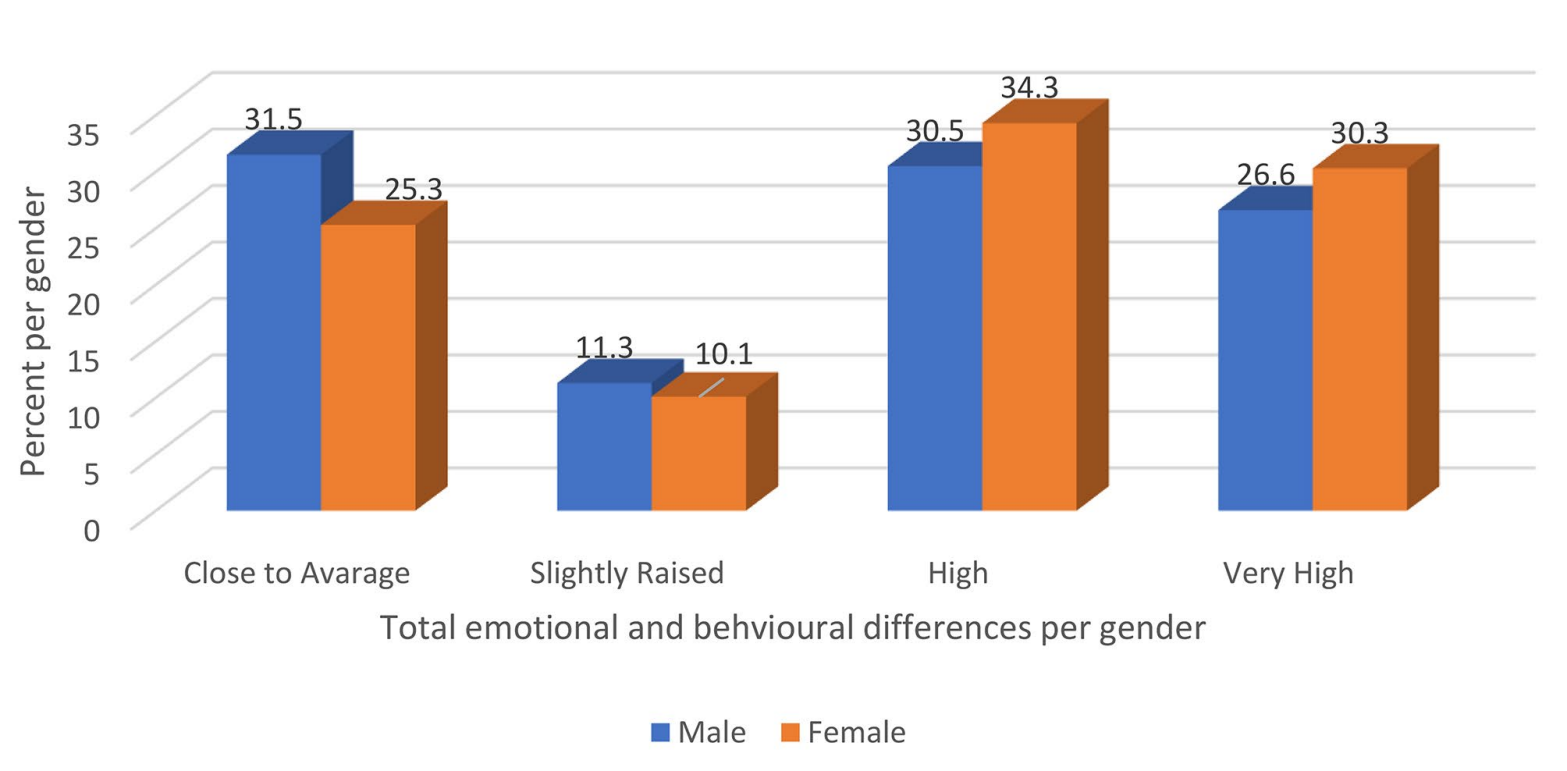
Gender differences in SDQ-Y scores based on the 4-band-categorisation

Distribution of total difficult scores based on four levels shows some trends by variable gender. Results show that within each level, males are having lower frequencies as compared to females. Among the four levels of total difficult scoring, level high has the highest frequency in general and by gender (See Fig. 1).

### Factors associated with emotional and behavioural difficulties of participants

Binary logistic regression was run to identify factors associated with the emotional and behavioural difficulties of participants of the children. Results from multivariate binary logistic regression indicate children and adolescents who were catholic (AOR 4.114; 95% CI:1.502–11.262, *p* = 0.006) or Pentecost (AOR 3.730; 95% CI: 1.155–12.043, *p* = 0.028) are more likely to have abnormal emotional and behavioural difficulties as compared to those children who were protestant. Results further demonstrate that children who do not have a single or both parents (AOR 3.404; 95% CI:1.563–7.416, *p* = 0.002) were more likely to have abnormal emotional and behavioural difficulties as compared to those with single or both parents. Lastly, results indicate that children who were in WHO HIV clinical stage 2 (AOR 2.536; 95% CI:1.005–6.395, *p* = 0.049) or 3 and 4 (AOR 8.459; 95% CI:1.5.820-10.544, *p* < 0.001) had more odds of having the mental disorder as compared with those children in WHO clinical stage 1.

**Table 3:** Factors associated with emotional and behavioural difficulties in simple and multiple binary logistic regression

Factors	Mental Health Status		Crude Odds Ratio		Adjusted Odds Ratio	
	Normal	Abnormal	OR[95%]	*P*-Value	AOR[95%]	*P*-Value
**Sex**						
Male	25(31.6)	54(68.4)	1		-	-
Female	50(25.3)	148(74.7)	1.370(0.773–2.429)	0.281	-	-
**Religion**						
Protestant	53(38.7)	84(61.3)	1		1	
Roman Catholic	10(18.9)	43(81.1)	2.713(1.257–5.855)	0.011	4.113(1.502–11.262)	0.006
Pentecost	8(16.7)	46(84.3)	3.155(1.371–7.259)	0.007	3.730(1.155–12.043)	0.028
Tradition	2(14.3)	12(85.7)	3.786(0.815–17.587)	0.089	2.130(0.399–11.379)	0.377
Muslim	2(10.5)	17(89.5)	5.363(1.191–24.155)	0.02	4.742(0.534–42.100)	0.162
**Participant’s Education Level**						
Up to primary	48(30.4)	110(69.6)	1		1	
Secondary and above	27(22.7)	92(77.3)	1.606(0.920–2.803)	0.096	1.632(0.747–3.566)	0.219
**Guardian’s Education Level**						
None	2(20.0)	8(80.0)	1			
Primary	23(31.9)	49(68.1)	0.533(0.105–2.710)	0.448	-	-
Secondary	44(30.3)	101(69.7)	0.574(0.117–2.812)	0.493	-	-
Tertiary	6(12.0)	44(88.0)	2.050(0.337–12.481)	0.436	-	-
**Guardian’s Occupation**						
Unemployed	44(27.0)	119(73.0)	1			
Employed	31(26.5)	86(73.5)	1.035(0.599–1.786)	0.903	-	-
**Single/both parents**						
Yes	42(33.3)	84(66.7)	1		1	
No	33(21.9)	118(78.1)	1.880(1.047–3.374)	0.034	3.404(1.563–7.416)	0.002
**WHO Clinical Stages**						
Stage 1	49(40.2)	73(59.8)	1		1	
Stage 2	16(23.5)	52(76.5)	2.222(1.088–4.538)	0.028	2.536(1.005–6.395)	0.049
Stage 3/4	10(11.5)	77(88.5)	6.556(2.752–15.615	< 0.001	8.459(5.820-10.544	< 0.001
Age			0.995(0.869–1.050)	0.341	-	-

## Discussion

To the best of our knowledge this is the first study to assess the emotional and behavioural difficulties of children and adolescents living with HIV who completed a disclosure process and were attending ART teen club in Malawi. The findings of the current study show that about three-quarters (72.9%) of the children and adolescents living with HIV had emotional or behavioural problems as revealed by self-rated using the SDQ-Y (Table 2). With regard to the SDQ-Y scoring categories, more than two-thirds of the participants had higher scores (slightly raised, higher, and very high) in the emotional (68.2%), conduct (68.6%), prosocial (57.8%) and peer relationship problem scales (62.8%) except for hyperactivity scoring which had (16.6%).

The higher score (slightly raised/ high/very high) of 68.2% for emotional problems found in the current study is surprising for this group of “ART teen club” as these participants would have been counselled and are aware of their diagnosis. One of the main benefit of disclosure of HIV status to HIV-infected children and adolescents is to enhance mental health status [33]. In Malawi, parents or guardians are encouraged to disclose their HIV status to their perinatally acquired HIV children gradually [34]. Perhaps reviewing to evaluate and revise the post disclosure counselling system can benefit the psychosocial wellbeing of this population. The high rate could also be attributed to participant’s everyday worries regarding their future, health as well as feelings of resentment an blame towards their caregivers [35]. Authors of previous studies have reported found few mental health challenges in children with full HIV status disclosure than those who were unaware of their HIV status [36, 37]. This calls for open dialogue about “teenage challenges” with a special focus mental health especially for those who are transitioning from a Teen Club to the adult clinic as they are at risk of attrition [34]. This is important because mental health ultimately influences retention in the HIV program and medication adherence [38].

The prevalence of conduct problems as measured by the SDQ-Y(68.6%) was extremely high in this study compared with 38% reported previously in selected facilities in Malawi [35]. This could be attributed to the sample size and the age of the participants recruited in these studies. The previously reported study recruited children as young as 6 years old and a parental version of SDQ was used to collect data. Differing results were also reported in Zambia (10.2%) [39] and the United Kingdom (3%) [40]. Similarly, the parent version of SDQ-Y was used in both studies. Children are well known to report more symptoms on SDQ-Y than parents [41]. Future studies should consider using the parental version of SDQ-Y as well to supplement data for an in-depth understanding of the subject and to make real time inferences. Even though there were a lot of children and adolescents of unemployed guardians (n = 119) compared to employed guardians (n = 86) who had emotional and behavioural problems in the current study (Table 3), the association did not reach the level of significance. This is in line with studies in developed and developing countries which reported that unemployed parents were more likely to report emotional and behavioural problems in their children compared to those who were employed [42, 43]. In addition, HIV in the family may escalate economic hardship as unemployment is increased and scarce family resources may be diverted to meet the needs of parents or guardians. In an earlier cross-sectional study done in Malawi and South Africa to explore cash grants on children’s cognitive development, results showed that helping families infected with HIV with cash receipts was associated with enhanced child cognitive outcomes [44]. Finding ways to assist socio-economically disadvantaged families with children and adolescents living with HIV with financial aid through social grants like cash transfers may reduce emotional and behavioural difficulties in these children and adolescents.

The current study has shown that children and adolescents who were in WHO HIV clinical stage 2 (AOR-2.536 95% CI, CL:1.005-6,395 *p* = 0.049) and stage 3 or 4 (AOR 8.459; 95% CI:1.5.820-10.544, *p* < 0.001) were more likely to score higher scores on SQD as compared with children in WHO HIV clinical stage 1. This finding is consistent with a previous study which reported that immunosuppression was associated with poor emotional and behavioural outcomes [45]. In a recent study done in Botswana, HIV congenitally infected adolescents were almost four times more likely to present with externalizing disorders like ADHD which may have occurred partly due to the effect of HIV on the brain [46] or immunosuppression resulting from prolonged period of poor ART adherence [47]. Researchers have found a strong association between externalizing disorders and viral load above 400 copies [46]. In another study children with low CD4 count < 500 cells/mm^3^ were more likely to develop emotional and behavioural difficulties as compared to children with CD4 count > 500 cells/mm^3^(*p* = 0.015) [48]. In a study by Kim, Mazenga, Yu et al., (2015), no significant correlation was found between mental health difficulties, and CD4 Count [49]. Rigorous longitudinal studies are therefore recommended to resolve the discrepancy as well are highlight the direction of the observed effect.

In this study caregivers’ level of education was not associated with the children and adolescents’ mental health challenges. Previous studies have reported caregivers with low levels of education had a higher likelihood of having children with a mental health problem [35]. In a study by Cortia, Fazel, Hlungwani et al. (2013) in rural Southern Africa mothers with lower educational levels (i.e. not completed high school) were more likely to report poor psychological outcomes for their children like hyperactivity, anxiety and learning difficulties [50]. Low education may also mean having low socio-economic status. Support for caregivers from socio-economic backgrounds is critical in a holistic treatment plan.

About half of the respondents (51.6%) of the children and adolescents in “ART Teen Club” in Mzuzu City had both parents. The results have shown that those who did not live with both parents had high chance of developing emotional and behavioural difficulties (AOR 3.404; 95% CI: 1.563–7.416, *P* = 0.002). Similar findings were found in other parts of Africa [51]. Research suggests that presence of parent is a protective factor for emotional problems. However, other researchers have recorded emotional problems in both double and non-orphans [52]. Perhaps a lack of socioeconomic support following the demise of parents or perceived collective family HIV- associated stigma magnifies the problem. Health workers in these clinics, should pay more attention to the orphans to timely identify and refer or manage emotional and difficult behaviours.

Our study has found that children and adolescents who were catholic (AOR 4.114; 95% CI:1.502–11.262, *p* = 0.006), Pentecost (AOR 3.730; 95% CI: 1.155–12.043, *p* = 0.028) or Muslim (4.742: 95% CI: 10.534–42.100, *p* = 0.02) are more likely to have abnormal emotional and behavioural difficulties as compared to those children who were protestant. This could be because of issues to do with spirituality or religiousness. Roman Catholics, Pentecost and Muslims are regarded to be of higher spirituality or religiousness than protestants. Several studies confirm our findings that low spirituality was associated with low emotional and behavioural challenges [53, 54]. For example, a study by Michealson, Robinson and Pickett (2014) in Canada found that Canadian youth with regular church involvement had poorer levels of emotional well-being compared to those who did not participate in religious services [54]. In contrast, a study by Lyona, D’Angeloa, Chengd et al., (2020) found that religiousness moderates adolescent medical decision-making. The authors recommended that religious adolescents should be encouraged to practice their religion to develop resilient and enhance mental well-being, which consequently, promote medication adherence [55].

## Strength of the study

The study used a well-established emotional and behavioural tool with strong psychometric properties validated for several African countries including Malawi. However, this tool heavily relies on self-report of children and adolescent, which has a potential bias of providing socially acceptable responses. In addition, this tool has been previously critiqued and is not recommended in children below 11 years [56]. Collecting data from other informants such parent and/or teacher would not only strengthen the rigour of the study, but also credibility of the emotional and behavioural difficulties evaluation [56]. The findings of the study should be interpreted with a consideration of limitations associated with cross-sectional studies such as limitation in determining casuality and direction of correlations.

## Implications of the study findings

The findings from this research study have several implications for practice, research, and policy, particularly in the context of child and adolescent emotional and behavioral difficulties.

### Implication for practice

In light of the study findings, there are several practical implications that merit consideration. Firstly, the Ministry of Health needs to intensify mental health screening in ART teen clubs as undiagnosed mental health problems may decrease antiretroviral therapy adherence leading to a lowering of immunity and acceleration of disease progression in these children and adolescents [38]. This approach facilitates the early identification of at-risk individuals, allowing for timely interventions and support. Secondly, healthcare providers operating in HIV clinical settings need to integrate mental health assessments and interventions. Implementing collaborative care models, involving both medical and mental health professionals, ensures a holistic approach to healthcare. This integration is crucial for promoting resilience by addressing both mental and physical health needs concurrently. Furthermore, given the association between abnormal emotional and behavioural difficulties and Catholic or Pentecostal affiliation, practitioners must integrate cultural and religious sensitivity into therapeutic approaches. For instance, incorporating spiritual practices within therapy for children and adolescents from these religious backgrounds may enhance engagement and improve outcomes. Lastly, interventions targeting children without one or both parents should concentrate on building robust support systems. Practitioners could collaborate with community organizations to establish mentorship programs, contributing to fostering resilience in children facing familial challenges. Strengthening protective factors, including supportive adult relationships, is vital for promoting resilience.

### Implications for Research

The study findings underscore the need for further research to deepen our understanding of risk factors contributing to emotional and behavioral difficulties. Qualitative studies exploring the lived experiences of children from diverse religious backgrounds can provide nuanced insights, informing targeted interventions and resilience-building strategies. In addition, longitudinal studies are imperative to track the developmental trajectories of emotional and behavioural difficulties in high-risk groups. Identifying critical periods for intervention contributes to resilience research by shedding light on factors that buffer against mental health challenges across the lifespan. Moreover, researchers should adopt an intersectional lens to explore the interplay of various risk factors contributing to emotional and behavioral difficulties. Investigating how religious affiliation intersects with familial structure, for example, provides a nuanced understanding. This approach contributes to resilience frameworks by identifying protective factors in diverse contexts.

### Implications for policy

Policy makers must consider allocating resources to implement evidence-based mental health programs targeting high-risk groups. Funding initiatives like Teen club and community-based mental health programs enhances accessibility and fosters resilience among children and adolescents, particularly those living with HIV. Furthermore, policies related to HIV care should explicitly integrate mental health components. Implementing guidelines that mandate routine mental health screenings within HIV clinical settings, including Teen clubs, ensures a comprehensive approach to healthcare. This aligns with resilience-oriented policies by addressing mental health needs alongside physical health. The findings of this study also underscore the need to formulate policies that specifically support vulnerable families, recognizing the unique challenges they face. Implementing financial assistance programs or community-based support services strengthens familial resilience. Policies aimed at supporting vulnerable families contribute to broader societal resilience.

## Conclusion

The findings of this research underscore the multifaceted nature of mental well-being among children and adolescents living with HIV. Elevated scores in total difficulties, emotional, conduct, and peer problems signify areas of concern, while disparities in hyperactivity and prosocial behavior highlight the nuanced nature of their behavioral challenges. Recognizing the inadequacy of a one-size-fits-all approach, the research emphasizes the necessity of a comprehensive strategy, incorporating factors like religious background, family structure, and clinical HIV stage. Furthermore, the role of Teen support clubs in this context is pivotal. Beyond addressing identified risk factors, these clubs must actively foster resilience. Creating an inclusive environment, tapping into individual strengths, and nurturing a sense of community are vital components. By adopting such a holistic approach, Teen support clubs can significantly contribute to the overall mental well-being of adolescents living with HIV, enabling them to navigate challenges effectively and thrive amidst their circumstances.

## Data Availability

The dataset used and/or analyzed during the current study are available from the corresponding author on a reasonable request.

## References

[1] Joint United Nations Programme on HIV/AIDS (UNAIDS). 2021. UNAIDS Data 2021. UNAIDS. [Internet]. Available from: https://www.unaids.org/sites/default/files/media_asset/JC3032_AIDS_Data_book_2021_En.pdf 12349391

[2] Bain R, Beise J, Benali N, Cappa C, Carvajal-Aguirre L, Diallo M et al. Progress for Every Child in the SDG Era. 2018; Available from: www.data.unicef.org.

[3] WHO. Guidance for HIV testing and counselling and care for adolescents living with HIV: Recommendations for a public health approach and considerations for policy-makers and managers. 2013; Available from: https://apps.who.int/iris/handle/10665/94334 25032477

[4] United Nations Programme on HIV/aids. UNAIDS. UNAIDS data 2021. 2021;4–38.

[5] SeyedAlinaghi SA, Mirzapour P, Pashaei Z, Afzalian A, Tantuoyir MM, Salmani R et al. The impacts of COVID-19 pandemic on service delivery and treatment outcomes in people living with HIV: a systematic review. AIDS Res Ther [Internet]. 2023;20(1):1–17. 10.1186/s12981-022-00496-7 10.1186/s12981-022-00496-7 PMC9821373 36609313

[6] Nassen R, Donald K, Walker K, Paruk S, Vujovic M, Duncan W (2014). Management of mental health disorders and central nervous system sequelae in HIV-positive children and adolescents. South Afr J HIV Med.

[7] Zgambo M, Arabiat D, Ireson D. “We just do it … we are dead already”: Exploring the sexual behaviors of youth living with HIV. 2022;(December 2021):34–44 10.1002/jad.12003 35353408

[8] Merikangas KR, Nakamura EF, Kessler RC. Epidemiology of mental disorders in children and adolescents. Dialogues Clin Neurosci [Internet]. 2009;11(1):7–20. Available from: 10.1001/jamapediatrics.2013.192 10.31887/DCNS.2009.11.1/krmerikangas PMC2807642 19432384

[9] World Health Organization (WHO). Comprehensive Mental Health Action Plan 2013–2030 [Internet]. Available from: https://www.who.int/publications/i/item/9789240031029 (Accessed 8 April 2023).

[10] Brandt R. The mental health of people living with HIV/AIDS in Africa: a systematic review. African J AIDS Res [Internet]. 2009;8(2):123–33. 10.2989/AJAR.2009.8.2.1.853 10.2989/AJAR.2009.8.2.1.853 25875564

[11] Vreeman RC, McCoy BM, Lee S (2017). Mental health challenges among adolescents living with HIV. J Int AIDS Soc.

[12] Ayano G, Solomon M, Abraha M (2018). A systematic review and meta-analysis of epidemiology of depression in people living with HIV in East Africa. BMC Psychiatry.

[13] Betancourt T, Scorza P, Kanyanganzi F, Smith Fawzi MC, Sezibera V, Cyamatare F et al. HIV and child mental health: a case-control study in Rwanda. Pediatrics. 2014;134(2). 10.1542/peds.2013-2734 PMC4187226 25049342

[14] Mellins CA, Xu Q, Nestadt DF, Knox J, Kauchali S, Arpadi S (2019). Screening for Mental Health among Young South African children: the Use of the strengths and difficulties Questionnaire (SDQ). Glob Soc Welf.

[15] Bankole KO, Bakare MO, Edet BE, Igwe MN, Ewa AU, Bankole IA et al. Psychological complications associated with HIV/AIDS infection among children in South-South Nigeria, sub-Saharan Africa. Cogent Med [Internet]. 2017;4(1):1372869. 10.1080/2331205X.2017.1372869

[16] Malee K, Williams PL, Montepiedra G, Nichols S, Sirois PA, Storm D (2009). The role of cognitive functioning in Medication Adherence of children and adolescents with HIV Infection. J Pediatr Psychol.

[17] Paramesparan Y, Garvey LJ, Ashby J, Foster CJ, Fidler S, Winston A. High rates of asymptomatic neurocognitive impairment in vertically acquired HIV-1-infected adolescents surviving to adulthood. Vol. 55, Journal of acquired immune deficiency syndromes (1999). United States; 2010. p. 134–6. 10.1097/QAI.0b013e3181d90e8c 20733406

[18] Bagenda D, Nassali A, Kalyesubula I, Sherman B, Drotar D, Boivin MJ (2006). Health, neurologic, and cognitive status of HIV-infected, long-surviving, and antiretroviral-naive Ugandan children. Pediatrics.

[19] Brahmbhatt H, Boivin M, Ssempijja V, Kagaayi J, Kigozi G, Serwadda D (2017). Impact of HIV and atiretroviral therapy on neurocognitive outcomes among school-aged children. J Acquir Immune Defic Syndr.

[20] Lentoor AG (2020). Clinico-immunological status and neurocognitive function of perinatally acquired HIV-Positive children on cART: a cross-sectional Correlational Study in South Africa. Front Neurol.

[21] Lyambai K, Mwape L (2018). Mental Health problems experienced by HIV positive adolescents; a case of Choma District, Zambia. Open J Psychiatry.

[22] Pettifor A, Stoner M, Pike C, Bekker LG (2018). Adolescent lives matter: preventing HIV in adolescents. Curr Opin HIV AIDS.

[23] Baylor College of Medicine International Pediatric AIDS Initiative (BIPAI). Baylor College of Medicine International Pediatric AIDS Initiative-Malawi Teen Club Curriculum Part 1: Content A Resource for Groups working with Adolescents Living with HIV. 2012;(February):1–76. Available from: https://toolkits.knowledgesuccess.org/toolkits/alhiv/bipai-malawi-teen-club-life-skills-curriculum (Accessed 2 April 2023).

[24] Management Sciences for Health. District Health System Strengthening and quality improvement for service delivery retain: Teen clubs help retain adolescents in HIV care and treatment. Mpawa H, Mchacha INC, Ngwalo C, Betha R, Birse S, Konings E, editor. [Internet]. Malawi: Management Sciences for Health. 2018. Available from: https://msh.org/wp-content/uploads/2018/02/cdc_-_teen_club_brief_revised.pdf

[25] Mackenzie RK, van Lettow M, Gondwe C, Nyirongo J, Singano V, Banda V et al. Greater retention in care among adolescents on antiretroviral treatment accessing Teen Club an adolescent-centred differentiated care model compared with standard of care: a nested case–control study at a tertiary referral hospital in Malawi. J Int AIDS Soc. 2017;20(3). 10.1002/jia2.25028 PMC5810310 29178197

[26] van Wyk BE, Davids LAC (2019). Challenges to HIV treatment adherence amongst adolescents in a low socio-economic setting in Cape Town. South Afr J HIV Med.

[27] Alibi M, Mwapasa V, Gwalangwa F. Retrospective cohort study comparing antiretroviral treatment outcomes among adolescents in Teen Clubs and Standard Care Clinics: Blantyre, Malawi. Res Sq [Internet]. 2023;1–15. 10.21203/rs.3.rs-2337996/v1%0ALicense 10.1177/23259582231172340 PMC10196531 37194291

[28] Novak D, Kawachi I (2015). Influence of different domains of social capital on psychological distress among Croatian high school students. Int J Ment Health Syst [Internet].

[29] Raosoft I. (2020). *Sample size calculator by Raosoft*. Retrieved 27/09 from http://www.raosoft.com/samplesize.html

[30] Goodman R, Meltzer H, Bailey V (1998). The strengths and difficulties Questionnaire: a pilot study on the validity of the self-report version. Eur Child Adolesc Pyschiatry.

[31] Hoosen N, Davids EL, de Vries PJ, Shung-King M. The Strengths and Difficulties Questionnaire (SDQ) in Africa: A scoping review of its application and validation. Child Adolesc Psychiatry Ment Health [Internet]. 2018;12(1):1–39. 10.1186/s13034-017-0212-1 10.1186/s13034-017-0212-1 PMC5765647 29344084

[32] Strengths and Difficulties Questionnaire. : Chichewa Version. Available from: https://sdqinfo.org/py/sdqinfo/b3.py?language=Chichewa

[33] Amankwah-Poku M, Klutsey DA, Asante KO. Disclosure and health-related outcomes among children living with HIV and their caregivers. AIDS Res Ther [Internet]. 2021;18(1):1–8. 10.1186/s12981-021-00337-z 10.1186/s12981-021-00337-z PMC8056491 33879193

[34] Malawi Ministry of Health. Clinical Management of HIV in Children and Adults. 2018 [Internet]. Available from: https://differentiatedservicedelivery.org/wp-content/uploads/malawi-clinical-hiv-guidelines-2018-1.pdf

[35] Kalembo FW, Kendall GE, Ali M, Chimwaza AF (2019). Prevalence and factors associated with emotional and behavioural difficulties among children living with HIV in Malawi: a cross-sectional study. BMC Psychiatry.

[36] Vreeman RC, Scanlon ML, Marete I, Mwangi A, Inui TS, McAteer CI et al. Characteristics of HIV-infected adolescents enrolled in a disclosure intervention trial in western Kenya. AIDS Care - Psychol Socio-Medical Asp AIDS/HIV [Internet]. 2015;27(00):6–17. 10.1080/09540121.2015.1026307 10.1080/09540121.2015.1026307 PMC4685612 26616121

[37] Abegaz BF, Walle TA, Tilahun AD. HIV positive status disclosure and associated factor among HIV infected children in pediatric ART clinics in Gondar town public health facilities, North West Ethiopia, 2018. J Infect Public Health [Internet]. 2019;12(6):873–7. 10.1016/j.jiph.2019.05.018 10.1016/j.jiph.2019.05.018 31213410

[38] Kim SC, Ecoff RNL, Brown NCE, Gallo CNSA, Stichler RJF (2017). Benefits of a Regional evidence-based Practice Fellowship Program: a test of the ARCC Model. Worldviews Evidence-Based Nurs.

[39] Menon A, Glazebrook C, Ngoma MS (2009). Mental Health of HIV positive adolescents in Zambia. Med J Zambia [Internet].

[40] Melvin D, Krechevsky D, Divac A, Tacconelli E, Miah J, Waugh S (2007). Parental reports of emotional and behavioural difficulties on the SDQ for school-age children with vertically acquired HIV Infection living in London. Psychol Heal Med.

[41] Van Roy B, Groholt B, Heyerdahl S, Clench-Aas J. Understanding discrepancies in parent-child reporting of emotional and behavioural problems: effects of relational and socio-demographic factors. BMC Psychiatry. 2010;10. 10.1186/1471-244X-10-56 PMC2912799 20637090

[42] Dockery A, Li J, Kendall G (2009). Parents’ work patterns and adolescent mental health. Soc Sci Med.

[43] Mendoza R, Hernandez-Reif M, Castillo R, Burgos N, Zhang G, Shor-Posner G (2007). Behavioural symptoms of children with HIV Infection living in the Dominican Republic. West Indian Med J.

[44] Sherr L, Macedo A, Tomlinson M, Skeen S, Cluver LD (2017). Could cash and good parenting affect child cognitive development? A cross-sectional study in South Africa and Malawi. BMC Pediatr.

[45] Ruiseñor-Escudero H, Familiar I, Nakasujja N, Bangirana P, Opoka R, Giordani B et al. Immunological correlates of behavioral problems in school-aged children living with HIV in Kayunga, Uganda. Glob Ment Heal. 2015;2. 10.1017/gmh.2015.7 PMC5269635 28596857

[46] Olashore AA, Paruk S, Akanni OO, Chiliza B. Psychiatric disorders in adolescents living with HIV in Botswana. AIDS Res Ther [Internet]. 2023;20(1):1–10. 10.1186/s12981-022-00490-z 10.1186/s12981-022-00490-z PMC9812345 36600270

[47] Rosenstein LD. ADHD as a Potential Risk Factor in Poor Antiretroviral Adherence Rates in HIV: A Brief Narrative Review and Suggestions for Future Research. Neuropsychol Rev [Internet]. 2021;31(4):683–8. 10.1007/s11065-021-09483-7 10.1007/s11065-021-09483-7 33580467

[48] Joshi D, Tiwari MK, Kannan V, Dalal SS, Mathai SS. Emotional and behavioral disturbances in school going HIV positive children attending HIV clinic. Med J Armed Forces India [Internet]. 2017;73(1):18–22. 10.1016/j.mjafi.2016.12.002 10.1016/j.mjafi.2016.12.002 PMC5221407 28123240

[49] Kim MH, Mazenga AC, Yu X, Devandra A, Nguyen C, Ahmed S et al. Factors associated with depression among adolescents living with HIV in Malawi. BMC Psychiatry [Internet]. 2015;15(1):1–12. 10.1186/s12888-015-0649-9 10.1186/s12888-015-0649-9 PMC4624356 26503291

[50] Cortina MA, Fazel M, Hlungwani TM, Kahn K, Tollman S, Cortina-Borja M et al. Childhood psychological problems in School settings in Rural Southern Africa. PLoS ONE. 2013;8(6). 10.1371/journal.pone.0065041 PMC3680478 23776443

[51] Cluver L, Orkin M, Boyes ME, Sherr L, Makasi D, Nikelo J. Pathways from parental AIDS to child psychologicducational and sexual risk: Developing an empirically-based interactive theoretical model. Soc Sci Med [Internet]. 2013;87:185–93. 10.1016/j.socscimed.2013.03.028 10.1016/j.socscimed.2013.03.028 23631794

[52] Doku PN (2009). Parental HIV/AIDS status and death, and children’s psychological wellbeing. Int J Ment Health Syst.

[53] Lyon ME, Kimmel AL, Cheng YI, Wang J, Medicine YA (2017). Of Life among adolescents with HIV: A Latent Profile Analysis. J Reli Heal.

[54] Michaelson V, Robinson P, Pickett W (2014). Participation in church or religious groups and its association with health: a national study of young canadians. J Relig Health.

[55] Lyona ME, D’Angeloa LJ, Chengd YI, Dallase RH, Garvie PA, Wang J (2020). The influence of religious beliefs and practices on health care decision-making among HIV positive adolescents. AIDS Care.

[56] Hall CL, Guo B, Valentine AZ, Groom MJ, Daley D, Sayal K et al. The validity of the strengths and difficulties Questionnaire (SDQ) for children with ADHD symptoms. PLoS ONE. 2019;14(6). 10.1371/journal.pone.0218518 PMC6583960 31216327

